# Immunisation status of children receiving care and support in Wales: a national data linkage study

**DOI:** 10.3389/fpubh.2023.1231264

**Published:** 2023-07-31

**Authors:** Grace A. Bailey, Alexandra Lee, Helen Bedford, Malorie Perry, Sally Holland, Suzanne Walton, Lucy J. Griffiths

**Affiliations:** ^1^Population Data Science, Swansea University Medical School, Swansea, United Kingdom; ^2^Population, Policy and Practice Research and Teaching Department, UCL Great Ormond Street Institute of Child Health, London, United Kingdom; ^3^Public Health Wales, No 2 Capital Quarter, Cardiff, United Kingdom; ^4^Children’s Social Care Research and Development Centre, School of Social Sciences, Cardiff University, Cardiff, United Kingdom

**Keywords:** vaccination, vaccine, immunisation, timeliness, children receiving care and support, looked after children, Child Protection Register, data linkage

## Abstract

**Background:**

In the UK, a robust childhood immunisation programme ensures children are offered protection against serious infections; identifying inequalities in vaccination coverage is essential. This is one of the first data linkage studies to examine coverage of primary, as well as pre-school booster and second dose of MMR vaccines, in children receiving support from social care services across Wales.

**Methods:**

By accessing records held within the Secure Anonymised Information Linkage (SAIL) Databank, vaccination status of children receiving social care and support between April 2016 and March 2021 (*n* = 24,540) was ascertained. This was achieved through linkage of the Children Receiving Care and Support (CRCS) Census and National Community Child Health Database which holds vaccination records for all children in Wales registered for NHS care. This sample was split into three groups – those children who had never been recorded on the Child Protection Register (CPR) or as ‘Looked After’ but in CRCS (*n* = 12,480), children ever on the CPR (*n* = 6,225) and those ever recorded as ‘Looked After’ but who were never on the CPR (*n* = 5,840). The comparison group of children and young people (CYP) never receiving welfare support consisted of 624,905 children.

**Results:**

Children receiving care or support were more likely to be up-to-date with all six vaccines (no recorded vaccines: 0.6–6.3%) compared to children in the comparison group (no recorded vaccines: 3–10.3%). However, of those who were vaccinated, they were less likely to be vaccinated in a timely manner; both early (5.2% vs. 22.2%; margin of error [ME] = 0.52, 95% CI [confidence interval] = −0.18 – −0.17, *p* < 0.001) and delayed vaccinations were more common (62.7% vs. 71.3%; ME = 0.58, 95% CI = 0.08–0.09, *p* < 0.001). Validation of the CRCS immunisation flag showed moderate levels of accuracy. Around 70% of immunisation flags were correct across all three groups.

**Discussion:**

Findings suggest a positive association between receiving services under a care and support plan and being up-to-date with immunisations; children receiving support under a care and support plan were more likely to have experienced early or late vaccinations, demonstrating that there is still more inter-disciplinary co-ordination and planning needed to improve these outcomes. Thus, identifying inequalities in vaccination coverage is essential to target interventions and to prioritise geographic areas for catch-up.

## Introduction

In the UK a robust childhood immunisation programme ensures that children are offered protection against serious infections (1). However, not receiving scheduled vaccines, or receiving them too early or late, not only leave children vulnerable to vaccine-preventable diseases but also compromises population (herd) immunity. Identifying and addressing factors associated with sub-optimal vaccine uptake may improve both vaccine timeliness, and overall vaccine uptake (2).

Children receiving support from statutory social care services are considered one of the most vulnerable groups in society, with high levels of unmet health needs (3, 4). In Wales, these children receive services under a Support and Care Plan. They comprise three main groups, with some overlap and movement between them: those who remain at home and whose names are on the Child Protection Register (CPR), children looked after (CLA) in foster care, kinship care or residential care, and all others with a Support and Care Plan who are neither looked after nor on the CPR (5).

More is known about the health of looked after children than those who receive support and care services at home. This is because looked after children receive statutory health assessments, whereas children receiving support services receive universal health services (6–8). Looked after children face a range of health challenges. Reasons identified for this include their adverse circumstances, such as neglect, poor parenting and challenging lifestyles which may have resulted in them entering the care system (3, 9). Instability of placements following admittance to care can lead to changes in primary care givers, schools and residential location, and hence contact with community services such as general practitioners (3), which may also be detrimental. These fractured pathways through health and care systems can result in routine health appointments being missed or not followed-up (10, 11).

There is also a concern that carers and social workers are not always provided with enough information about the health history of children when entering care (12). Therefore, they may not be aware of potential conditions requiring medication or intervention from health professionals.

A more specific issue concerning the health of children in care is their immunisation status. Barnes et al. (9) suggest that incomplete immunisation of children entering care may reflect the health neglect experienced by these children. More recent, although limited evidence consistently suggests children in care are less likely to have a full and up-to-date immunisation status than their general population peers (4, 13–15). Research evidence specifically about children on the Child Protection Register (CPR), and those receiving support from social services, is even more limited (16).

Despite yearly increases in the number of children receiving care and support in Wales (17), of the few UK-based studies described above, these have been limited to local authorities. There are very few large-scale studies examining immunisation status within this population at a nationwide level. Reporting across the whole of Wales is particularly important as vaccination coverage varies by region (18). More recently conducted studies are also required to examine the effects of changes to government legalisation and policies on immunisation receipt and timeliness (19). Detailed information about the immunisation status amongst this vulnerable cohort also has the potential to improve area-based services and immunisation coverage, as well as identifying any hard to reach groups (20).

Within Wales, the Children Receiving Care and Support (CRCS) Census collects individual records annually on all children who have a care and support plan. The purpose of the Census is to collect data on the characteristics and attributes of children receiving social support from social services, including children looked after by local authorities. The Census focuses on the reason for children receiving support from social services departments, parental capacity, and on the health and education outcomes for each child (21). This includes those looked after by a local authority, who in each year, had an open case since 1st January of each year (21). It also flags child protection cases (those on the Child Protection Register (CPR) who have been identified as being at risk of harm or experiencing harm) and children ‘looked after’ by the local authority (CLA), sub-groups of the CRCS cohort who are subject to more intensive services (16).

Based on this information, statistics on immunisation status are published (22), showing for example, that in 2020, immunisation information was available for 96.8% of those in the Census, of whom 91.7% (*n* = 14,720) were recorded as ‘up-to-date’ across Wales. However, to our knowledge, completeness, and timeliness of vaccination for children receiving care and support has not been documented, nor has the validity of this flag been checked against routine immunisation programme records. Routine vaccination records for children up to 18 years of age, are recorded in the Wales National Community Child Health Database (NCCHD) population register, which is used to produce national vaccination coverage figures (18). The NCCHD data are extracted from local child health systems, which are used locally for scheduling and sending out vaccination appointments.

This study therefore linked Welsh CRCS Census records to routine child health vaccination records held in the NCCHD. The primary objective of this study was to establish the timeliness of vaccine receipt, with the aim of understanding the prevalence of a selection of delayed primary and pre-school vaccines [first, second and third dose of diphtheria/tetanus/pertussis (DTP), first dose of Measles, Mumps and Rubella (MMR), as well as pre-school DTP booster and second dose of MMR (MMR2) vaccine] in this population compared to a group of children never having received care and support; the second objective was to examine the CRCS immunisation status flag and NCCHD vaccination records side-by-side to assess validity of the flag.

## Methods

### Study design

This was a secondary data analysis of a population-level cohort study, with the group of interest being children receiving social care and support.

### Study population

**Figure 1:**
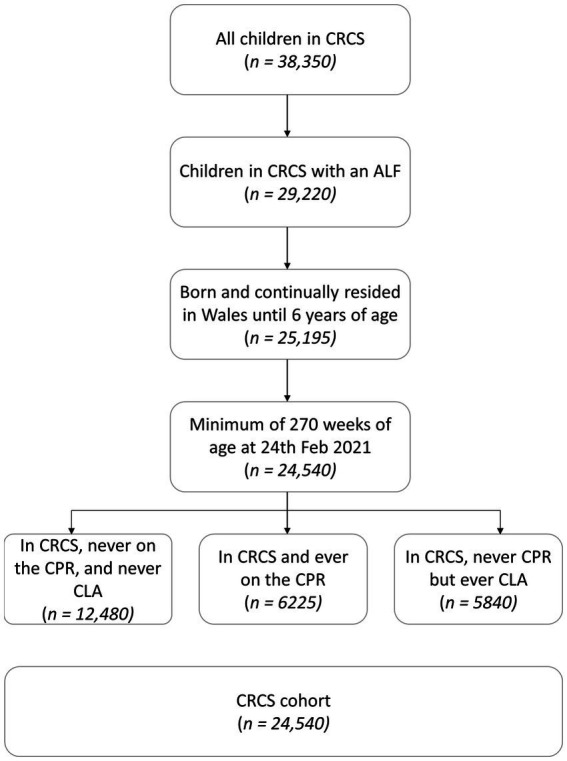
Consort creation flow chart.

The study sample included all children recorded as receiving care and support in the CRCS census between 1st April 2016 and 31st March 2021, with an anonymised linking field (ALF; see below), who were born and continually resided in Wales until 6 years of age (*n* = 24,540) (Figure 1). Children in the study sample were required to be of an age to have received all the specified vaccines and for these records to be available in the National Community Child Health Database (NCCHD); meaning that they had to be a minimum of 270 weeks (5.2 years) of age on 24th February 2021. Children can appear in the CRCS census up until their 18th birthday. Children without a valid ALF could not be linked to vaccination records in the NCCHD.

This sample was split into three groups (Figure 1) – those in the CRCS Census but never recorded as on the CPR or as ‘Looked After’ [‘never CPR or CLA’ (*n* = 12,480)], children on the Child Protection Register ‘ever on CPR’ (*n* = 6,225), those ever recorded as ‘Looked After’ but who were never on the CPR ‘ever CLA’ (*n* = 5,840).

In addition, this study utilised a population comparison group of children born and continually residing in Wales, who had never appeared in the CRCS, were of an age to have received all the specified vaccines (at least 270 weeks of age on 24th February 2021) and with records in the NCCHD (Appendix 1). To ensure parity with the study sample we also required that all children in the comparison group were the same age or younger than the oldest child in the CRCS cohort (June 1996).

### Data sources and linkage

Data were obtained via the SAIL (Secure Anonymised Information Linkage) Databank, (23–25) which contains extensive anonymised health and administrative data about the population of Wales, accessible in anonymised form via a secure data sharing platform, all underpinned by an innovative and proportionate Information Governance model. All data within the SAIL Databank are treated in accordance with the Data Protection Act 2018 and are compliant with the General Data Protection Regulation.

Personal identifiable data were not used in this study. During the anonymisation process of data sources within the SAIL Databank, individuals are assigned an ALF based on their National Health Service (NHS) number, name, sex, date of birth and postcode. This anonymisation and linkage methodology has previously been described (25). Use of an ALF for each child enabled linkage of the following datasets: (a) the CRCS Census for years 2016 to 2021, with the all-important immunisation flag for this study. This data collection was previously named the Children in Need Census, changing to CRCS after the Social Services and Well-being (Wales) Act 2014 came into force in April 2016, to reflect better the children eligible for inclusion. The Social Services and Well-being (Wales) Act 2014 outlines the statutory duties local authorities are required to follow when supporting children in need of children’s social services (26); (b) the NCCHD, which brings together data from local child health system databases held by NHS organisations and includes information from birth registrations, child health examinations and vaccinations. Date of birth (week of birth) and date of vaccination were used to calculate age at vaccination. To protect an individual’s identity, whilst allowing a sufficient level of detail only week of birth is available. A total of 9,630 children in CRCS were excluded due to a lack of ALF. It is possible these children have different demographics compared to those with an ALF, however, we have not compared for potential bias. Due to our cohort eligibility rules, we used NCCHD information for years 1996 to 2018; and (c) the Welsh Demographic Service Dataset (WDSD), from 1990 to 2021, which provides demographic characteristics of people registered with a general practice (GP; doctor) in Wales, providing demographic and address details. We calculated deprivation using Lower Layer Super Output Area at the point involved with social services (at Census date) and at the point they should have had vaccinations.

### Main outcome measures

#### Timeliness of vaccination

Vaccination schedules (27) for the UK have changed repeatedly over the years. Our cohorts should have received routine vaccinations as shown in Table 1. DTP is usually given in combination with other vaccines. From 1996 it was given as a 4-in-1 vaccine (DTP-Hib), this was replaced in 2004 with the 5-in-1 (DTaP/Hib/IPV), and replaced with a 6-in-one vaccine (DTaP/Hib/IPV/HepB) in 2017 (29). Due to difference in age, our cohort could have been vaccinated with the 4-in-1, 5-in-1 or 6-in-1, therefore we considered DTP separately. Timeliness of vaccination was classified as early, on-time, delayed, or never, based on the recommended vaccination schedule. For the primary vaccines (first, second and third dose of DTP) we defined a vaccine as being given ‘on time’ if given in the interval between the age when the vaccine was due and the age when the next dose was due; ‘early’ as being given prior to these ages; and ‘delay’ when given after the latest ‘on time’ ages (Table 1) (28). For the first and second dose of MMR and the DTP pre-school boosters, ‘on time’ was defined as 12–15 months and three years four months to five years, respectively. Missing vaccine records were taken to mean unvaccinated.

**Table 1:** Vaccine schedule and definitions used for timeliness of vaccinations.

Vaccines	Due at age	Timeliness of vaccination based on age at which vaccine received			
		Early	On time	Delayed	Never
Primary vaccines					
DTP 1	8 weeks	<8 weeks	8–12 weeks	>12 weeks	Not at all
DTP 2	12 weeks	<12 weeks	12–16 weeks	>16 weeks	Not at all
DTP 3	16 weeks	<16 weeks	16–20 weeks	>20 weeks	Not at all
MMR 1	1 year	<12 months	12–15 months	>15 months	Not at all
Pre-school booster and MMR2					
DTP and MMR2	3 years, 4 months	<3 years, 4 months	3 years, 4 months to 5 years	>5 years	Not at all

DTP, Diphtheria, Tetanus and Pertussis; MMR, Measles, Mumps and Rubella.

Adapted from: Walton et al. (28).

#### Immunisation status

Children receiving statutory care and support should have regular reviews of their support, health and care needs, including a review of their immunisation status; where observed, deficiencies should be addressed. The CRCS Census flag for immunisation ‘up-to-date’ was one of the studies main outcome measures. This is defined as ‘up-to-date’ if the child has had all immunisations that a child of that age should have received by 31 March 2021 according to current information by Public Health Wales (30). Even if immunisations have been given late, the child will been considered as brought ‘up-to-date’. Assessing whether a child’s immunisations are ‘up-to-date’ is a clinical decision by a doctor or practice nurse (31).

As a secondary outcome, we examined the validity of the immunisation status, information relation to immunisations including type of vaccine and date the vaccine was administered was obtained from the NCCHD dataset.

### Statistical analysis

Data preparation was performed in Structured Query Language (SQL) on an IBM DB2 platform. Data were analysed using R software (version 1.4.3), chi-squared tests were utilised to identify any proportional differences in the timeliness of vaccinations amongst those receiving care and support and the comparison group. Margin of error (ME), 95% confidence intervals (CIs) and *p*-values for proportional differences are reported. Bonferroni correction was applied to adjust for multiple comparisons.

## Results

### Immunisation status as recorded in CRCS

There were 38,350 children receiving care and support included in the CRCS Census between 31st March 2016 and 31st March 2021 years (Table 2). Of these, 20.5% had ever had a ‘not-up-to-date’ flag for immunisation status. Additionally, around a quarter of the full CRCS cohort (26.5%) had been ‘ever CPR,’ of which 5.8% ever had a ‘not-up-to-date’ flag. Finally, 4.6% of all children and young people (CYP) included in the CRCS Census were ever CPR and ‘not-up-to-date’ at the same time.

**Table 2:** Immunisation status of children receiving care and support based on ‘up-to-date’ flag.

	n (%)
All children in CRCS 2016–2021	38,350
Ever ‘not-up-to-date’	7,845 (20.5)
Ever on CPR	10,160 (26.5)
Ever on CPR + ‘not-up-to-date’	2,210 (5.8)
Ever on CPR + ‘not-up-to-date’ at the same time	1,780 (4.6)
Study Cohorts for linkage work to health records	
All children in CRCS cohort	24,540
Ever ‘not-up-to-date’	5,145 (21.0%)
All children in CRCS cohort ever on CPR	6,225
Ever ‘not-up-to-date’	1,330 (5.4%)
All children in CRCS cohort never on CPR and never CLA	12,480
Ever ‘not-up-to-date’	2,445 (10.0%)
All children in CRCS cohort ever CLA but never CPR	5,840
Ever ‘not-up-to-date’	1,370 (5.6%)

The linked cohort (*n* = 24,540) comprised of 13,260 (54%) males and 11,280 females (46%) with a median age of 10 years. Similarly, the comparison group consisted of 320,305 males and 304,600 females (51.3 and 48.7%, respectively) median age of 14 years. Around a fifth (21.0%) of children in the CRCS cohort had a ‘not-up-to-date’ flag. 5.4% of those in the CRCS cohort ever on the CPR had this same flag, compared to 10% of all children in the CRCS cohort never on the CPR or CLA.

### Timeliness

**Figure 2:**
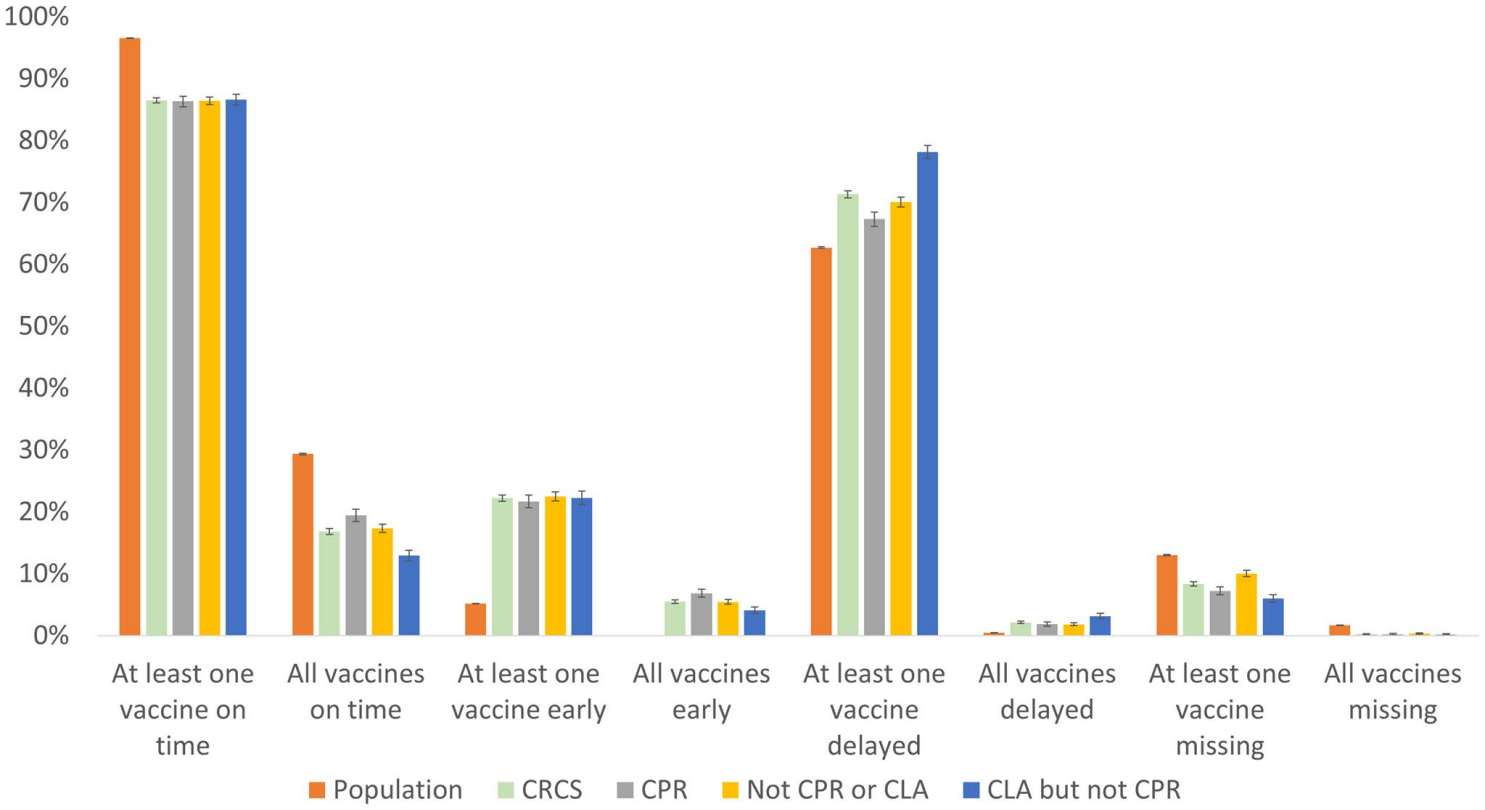
Timeliness of vaccines in the population and CRCS cohorts. Error bars represent 95% CIs for proportions.

Linkage of social care and NCCHD records enabled exploration of timely, early, delayed or no-immunisation status for a selection of infant and pre-school booster vaccines (Figure 2 and Appendix 2). The number of children who received at least one vaccine on time was comparable between the three CRCS cohorts (86.3–86.6%; CPR and not CPR/CLA: ME = 1.04, 95% CI = −0.01 – 0.01, *p* = 0.89; CLA and not CPR/CLA: ME = 1.06, 95% CI = −0.01 – 0.01, *p* = 0.68; CPR and CLA: ME = 1.22, 95% CI = −0.02 – 0.01, *p* = 0.63), although significantly lower than in the comparison population cohort (86.5% vs. 96.5%, ME = 0.43, 95% CI = 0.01–0.1, *p* < 0.001). Only a fifth (19.4%) of children in the CPR cohort received all their vaccines on time, with 17.3% of those never in the CPR or CLA, but never on CPR or CLA, and 12.9% CLA but never CPR, having all vaccines on time.

The percentage of those receiving at least one vaccine early was similar between the study cohorts (21.7–22.5%; CPR and not CPR/CLA: ME = 1.26, 95% CI = −0.02 – 0.005, *p* = 0.22; CLA and not CPR/CLA: ME = 1.29, 95% CI = −0.02 – 0.22, *p* = 0.74; CPR and CLA: ME = 1.48, 95% CI = −0.01 – 0.02, *p* = 0.45), with rates highest amongst those ‘never in the CPR nor CLA’ cohort and lowest in the ‘ever CPR’ cohort. Interestingly, early vaccinations were less likely in the comparison population group compared to the CRCS cohort (5.2% vs. 22.2%, ME = 0.52, 95% CI = −0.18 – −0.17, *p* < 0.001). Children within the CRCS census were more likely to receive at least one delayed vaccination in comparison to the population group (71.3% vs 62.7%, ME = 0.58, 95% CI = 0.08–0.09, *p* < 0.001). Further, at least one delayed vaccination was most common amongst the ‘ever CLA’ cohort (78.2%), 70.1% of those ‘never CPR nor CLA’, followed by children in the ‘ever CPR’ cohort (67.3%) and least likely to occur in the population cohort (62.7%).

Finally, those in the population cohort were significantly more likely to have vaccines missing (8.4% vs 13% at least one: ME = 0.36, 95% CI: −0.05 – −0.04, *p* < 0.001; all missing 0.3% vs 1.7%, ME = 0.07, 95% CI = −0.01 – −0.01, *p* < 0.001) than those within the CRCS cohorts.

### Missing vaccines

Children included in all three of the CRCS cohorts were less likely to have missing vaccines than those within the population comparison group (Table 3).

**Table 3:** Proportion of children born between 1996 and 2016 with no recorded vaccination in the NCCHD as at 24/02/2021.

	Population cohort	CRCS cohort	Never CPR nor CLA	Ever CPR	Ever CLA
DTP 1	18,980 (3.0%)	255 (1.0%)	160 (1.3%)	35 (0.6%)	60 (1.0%)
DTP 2	22,100 (3.5%)	320 (1.3%)	195 (1.6%)	55 (0.9%)	70 (1.2%)
DTP 3	25,055 (4.0%)	415 (1.7%)	235 (1.9%)	85 (1.4%)	90 (1.5%)
DTP booster	55,195 (8.8%)	950 (3.9%)	605 (4.8%)	230 (3.7%)	115 (2.0%)
MMR1	41,460 (6.6%)	780 (3.2%)	470 (3.8%)	170 (2.7%)	140 (2.4%)
MMR2	64,350 (10.3%)	1,180 (4.8%)	785 (6.3%)	250 (4.0%)	145 (2.5%)

Similar patterns of suboptimal immunisation status were observed between the population cohort and all three CRCS cohorts. Children were most likely to be unvaccinated with MMR2, including over 10.3% in the population cohort and 2.5–6.3% in CRCS cohorts, whilst DTP 1 vaccines were least likely to be missing (0.6–1.3% CRCS cohorts vs. 3% population group). Closer inspection of the CRCS cohorts shows that missing vaccines were comparable between all three groups (up to 0.7% variation) for DTP 1–3 vaccines, with higher fluctuations for DTP booster, MMR1 and MMR2 vaccines (up to 3.8%).

### ‘Validation’ of CRCS immunisation flag within the social care records

Linkage of the ‘up-to-date’ flag and NCCHD immunisation records revealed that approximately three-quarters of all CRCS cohorts had correct (70.1–77.2%) immunisation flags (Table 4). To validate immunisation flags, we compared immunisation flags to immunisation status at the time flags were added for each CRCS collection that the child was recorded in.

**Table 4:** ‘Validation’ of CRCS immunisation flag.

	CRCS cohort	Never CPR nor CLA	Ever CPR	Ever CLA
Total n (rows)	56,715	22,330	13,740	20,650
n Flag correct	41,825 (73.7%)	15,660 (70.1%)	10,235 (74.5%)	15,935 (77.2%)
n Flag incorrect	14,890 (26.3%)	6,670 (29.9%)	3,505 (25.5%)	4,715 (22.8%)
n marked up-to-date; child is not	9,215 (16.2%)	4,025 (18.0%)	2,140 (15.6%)	3,050 (14.8%)
n marked not up-to-date; child is	5,675 (10.0%)	2,645 (11.8%)	1,365 (9.9%)	1,665 (8.1%)
Where immunisation flag is not accurate, vaccines that are missing				
MMR1	1,415 (9.5%)	715 (10.7%)	325 (9.3%)	375 (8.0%)
MMR2	1,865 (12.5%)	1,190 (17.8%)	355 (10.1%)	325 (6.9%)
DTP 1	6,425 (43.1%)	2,555 (38.3%)	1,535 (43.8%)	2,335 (49.5%)
DTP 2	4,800 (32.2%)	1,990 (29.8%)	1,145 (32.7%)	1,665 (35.3%)
DTP 3	3,680 (24.7%)	1,535 (23.0%)	850 (24.3%)	1,295 (27.5%)
DTP booster	1,540 (10.3%)	925 (13.9%)	330 (9.4%)	285 (6.0%)
Incorrect records per collection year				
2016/17	3,255 (27.8%)	1,630 (31.5%)	550 (25.3%)	1,075 (24.7%)
2017/18	3,375 (28.9%)	1,495 (33.3%)	780 (28.6%)	1,100 (24.7%)
2018/19	3,395 (29.2%)	1,465 (33.0%)	835 (28.9%)	1,100 (25.6%)
2019/20	2,540 (22.3%)	1,130 (26.5%)	655 (21.3%)	755 (18.6%)
2020/21	2,325 (22.5%)	955 (24.1%)	685 (23.9%)	685 (19.6%)
Total number of records per collection year				
2016/17	11,710 (20.6%)	5,175 (23.2%)	2,175 (15.8%)	4,360 (21.1%)
2017/18	11,675 (20.6%)	4,495 (20.1%)	2,730 (19.9%)	4,450 (21.5%)
2018/19	11,630 (20.5%)	4,445 (19.9%)	2,890 (21.0%)	4,295 (20.8%)
2019/20	11,390 (20.1%)	4,260 (19.1%)	3,080 (22.4%)	4,050 (19.6%)
2020/21	10,315 (18.2%)	3,955 (17.7%)	2,865 (20.9%)	3,500 (16.9%)

Children not in CPR or CLA were most likely to have incorrect immunisation flags on their records (29.9%), with 18% marked ‘up-to-date’ although the child was not and potentially 11.8% marked ‘not up-to-date’ despite the child being up-to-date. In contrast, those in the CLA but not the CPR group were least likely to have incorrect flags. Again, an increase in errors occurred with the child being marked ‘up-to-date’ when the child was not (14.8%) compared to being marked ‘not up-to-date’ when the child was (8.1%).

When immunisation flags were inaccurate, vaccines were more likely to be missing for DTP 1–3 vaccines, with DTP 1 missing vaccines peaking at nearly half (49.5%) of the CLA but never CPR cohort. MMR 2, closely followed by MMR 1 vaccines were least likely to be missing where flags were inaccurate (8–10.7%).

Between all three groups, older records (2016/2017; 24.7–31.5%) were more likely to be incorrect compared to newer records (2020/2021; 19.6–24.1%).

## Discussion

To our knowledge, this is the first nationwide population-based cohort study to use linked cohort data to investigate the uptake and timeliness of childhood vaccinations in children receiving care and support in Wales.

Around one fifth of children receiving care and support were ever recorded as ‘not-up-to-date’ on the CRCS Census. Interestingly, the proportion of children receiving care and support who had had none of the six vaccines, was lower than in the comparison population cohort. Although children receiving care and support were less likely to be in receipt of at least one vaccine in a timely manner compared to the population group, they were more likely to receive at least one vaccine early or delayed in comparison to the population group. Finally, we demonstrate the accuracy and use of immunisation flags in the CRSCS dataset as a powerful tool to monitor uptake and timeliness of vaccines. Validation of immunisation flags showed around 70% of immunisation flags were potentially correct.

Some previous studies have examined immunisation rates of looked after children in comparison with the general population, but few have included other groups of children receiving statutory social work services (21). Of the few studies that have focused on immunisations in children ‘looked-after,’ previous work has consistently demonstrated that this population are less likely to be up-to-date with their immunisations compared to children not in care (4, 9, 32, 33), with the exception of one study which reported that children ‘looked-after’ are more likely to be fully immunised against MMR (84%) compared to not ‘looked-after’ children (80.8%) (34). However, it is important to note that a direct comparison between looked-after children and the general population is not possible because there is no comparable ‘up-to-date’ figure for all CYP. It is only possible to compare uptake of specific antigens by age.

In contrast to most previous work, we found that children receiving care and support were less likely to be missing DTP, DTP boosters and MMR vaccines compared to the Welsh population cohort.

It is difficult to hypothesise the potential reasons for this, as this finding largely holds across the three categories of children receiving care and support, despite their different levels of state intervention. Looked after children have usually been placed in foster or kinship care and receive statutory health assessments where their immunisations are reviewed. In contrast, children on the CPR will usually be living at home in conditions where they are experiencing or are likely to experience abuse or harm. Children receiving care and support who are not on the CPR nor looked after will usually be living at home in a wide range of circumstances, including child disability and with parents struggling to cope. The one consistent factor between the three groups is that they have allocated social workers and hence, potentially, an additional professional in their lives to remind and prompt responses to immunisation invitations. These social workers are likely to be prompted to discuss immunisations with the family due to the requirement to record immunisation status on care and support records. However, limited available information makes it difficult to draw any further conclusions. Improvements in GP guidelines, data collection, changes to service planning and amendments to policies are factors likely to have contributed to increased vaccine coverage over time.

Interestingly, when a vaccine was given, children receiving care or support were more likely to receive it early or delayed in comparison to children in the population group who were more likely to receive vaccines on time. Prolonged delays within this vulnerable population are consistent with previous findings and may reflect challenges surrounding the need for parental consent or lack of coordinated care (4, 18). Children ‘looked-after’ are a highly mobile population, making it challenging to maintain accurate health records as they move across different local areas or from other areas in the UK and change GP (35). Lack of stability is thought to be a major contributory factor to incomplete immunisation, resulting in lack of continuity in primary care. This is particularly evident in the first year of life when the primary vaccine course is offered, likely contributing to the low-uptake seen within this cohort, but is also an important cause of missed school based vaccination (16). These previously recorded instabilities likely explain the lack of timeliness with this cohort in our study, but, encouragingly, the overall picture appears to be that this cohort catch up and exceed the rest of the population by the age of six, suggesting a potential protective impact of becoming ‘looked-after.’

### Strengths and limitations

The main strength of this study was the large sample size identified by using routinely collected data. The nature of the data also removes the risk of discrepancies or errors in parental recall, while allowing for longitudinal monitoring of vaccine uptake. Data quality was further strengthened by combined use of data from the national CRCS and NCCHD datasets, increasing the likelihood that these finding can be extrapolated to all children receiving care or support in Wales.

As with any data linkage study there are limitations. Such data are not collected with the primary intention of being used for research and their quality may differ between individuals and local health boards. As a result, it may be the data that are incorrect or incomplete rather than a lack of immunisation, or vice versa. For example, it is possible that a vaccine could be given by a GP and that information not notified to the NCCHD or children from overseas or elsewhere in the UK may be given vaccines in other locations which can be challenging to record. This suggestion is supported by the frequency of potentially inaccurate immunisation flags within the CRCS census dataset (~30%) and demonstrates the need for improved communications between health and social services or development of electronic IT solutions to ensure records are as up to date as possible. Communication of health data to those with parental responsibility is also a key issue for looked-after children, further complicated by the need to obtain their consent for immunisation. It is plausible that the length of time children are in the care of the local authority may also affect vaccine uptake and timeliness. Future work should investigate longitudinal differences in immunisation coverage, for example when the child is taken into care compared to when they are established in care. The reasons for children not being up-to-date with vaccinations are well established, however, there may still be a need to explore this through qualitative research among specific groups such as children receiving care and support. In addition, qualitative research could also explore enablers and barriers to immunisation coverage for children receiving care and support, this would support our understanding of the patterns observed in this study.

To protect an individual’s identity, only week of birth is available in the datasets. It is possible that lack of precision in calculating the exact age at immunisation may affect estimates of timeliness, particularly in the first dose of DTP. Our analysis was based on several years’ worth of data, it is therefore possible that there may be differences in yearly trends. We only focused on six childhood vaccines, other routine vaccines such as rotavirus were not included, and therefore we are unable to say if children were truly up-to-date. Additionally, our analysis was limited to those who were born and lived in Wales up to 6 years of age. Variations in the definition of immunisation timeliness make it difficult to compare findings across studies, especially among countries which follow different vaccine schedules. To address this, we used definitions previously published (28). Finally, although we have speculated that delay in immunisation may be caused by lack of stability, it was not possible to explore the reasons for immunisation delay. Intentional delays in vaccines are not uncommon, up to 21.8% of US parents report intentionally delaying vaccines, mainly due to concerns around efficacy or safety (44.8%) (36). To overcome this, it is important that policies are put in place to encourage timely vaccination.

### Implications for policy and practice

#### Health assessments and care plans

There is substantial evidence that children ‘looked-after’ have extensive unmet health needs (3). To address this, statutory guidance was amended, and requires that every child coming into care receives a statutory initial health assessment with a paediatrician or health visitor and thereafter every 6 months up to the age of five. Those over 5 years of age have an annual health review (37) NICE guidelines (National Institute for Health and Care Excellence) recommend vaccinations should be part of a CYP initial health assessment and annual reviews (38, 39). It is the responsibility of the Local Authority to ensure that health assessments are completed in a timely manner and that all health information is accurate and up-to-date. Local authorities should have a system in place to monitor and review the care plan, this is vital to ensure that children’s health needs are met and the long-term risks are minimised (40). Performance monitoring is also likely to drive improved compliance with health assessments (41).

Despite the uptake of initial health assessment exceeding 90% (42), a study from 2009 found only just over half of health recommendations were being implemented. Identified barriers included incorrect GP details, difficulties in obtaining family histories and problems ensuring immunisations were up-to-date (43). A more recent literature review suggests that a more comprehensive health assessment could increase likelihood of specialist referral and therefore improve chances of healthcare interventions (44). Here, we find CLA (5.6%) and children on the CPR (5.4%) were less likely not to be up-to-date than children in CRCS (10%), likely due to these additional health interventions for looked after children, and the close, multi-disciplinary monitoring of those on the CPR.

#### Looked after children’s nurses

Discontinuity of care is a well-established barrier to immunisation among vulnerable children (33). To improve continuity and communication, services overseen by specialised nurses with additional experience and expertise in looked-after children (LAC) were introduced. The primary role of the LAC nurse is promoting the health and wellbeing of children in care, ranging from checking immunisation status to individualised services (45). Detailed health care plans are formulated with specific recommendations, e.g., obtaining history, requesting past medical history or arranging referrals. In Scotland, improved communication between health and social services facilitated by specialist nurses, improved immunisation rates among looked-after children (46). In England and Wales, due to low uptake of outstanding vaccines, unaccompanied minors are now referred to LAC nurses who liaise with GP surgeries or the school nurse (e.g., for HPV or school leaver boosters) to ensure immunisation courses are completed (43). It is the role of social workers to ensure that a plan and adequate arrangements are made, e.g., arrange parental consent for immunisations (37). Where immunisation status is incomplete or unknown, especially in unaccompanied minors, the uncertain immunisation schedule (UKHSA) is recommended (47). Here, we demonstrate that such improved policies and procedures may be having a positive effect on the uptake of vaccinations for this cohort.

#### Interdisciplinary collaborations

Clinical practice must remain receptive to the changing needs among children ‘looked-after’ and those receiving care and support services at home. Regular auditing of health is necessary to allow for effective planning of appropriate services (48). In turn, this would aid the Local Authority and care providers in the provisioning of appropriate access and delivery of high-quality multi-agency services. Working as a multidisciplinary team comes with its own challenges but is vital to meet the multifaceted needs of children receiving care and support. The complexity of the task requires co-ordinated and effective sharing of information between health and social services to improve access to services. However, the lack of protocols and combined health and social services databases can have major implications for the care of children from vulnerable groups. Often there are gaps in administrative recording of health data which can result in missing immunisation records or poor coverage. A further complication is that, for Welsh looked after children, nearly a third of children are placed outside their home local authority boundaries, contributing to additional delays in the health services provided (49).

Several government-led initiatives to encourage integrating services for vulnerable children to enhance their wellbeing have been introduced. The Welsh Community Care Information System (WCCIS), established in 2015, provides a unique opportunity to support community nurses, mental health teams and social workers (50). It aims to facilitate ease of communication across health and social services, as well as regional and organisational boundaries. Not only does this system allow for integration of information which can be shared more readily, it also has the potential to improve record-keeping of immunisation status for children receiving care and allow for appropriate planning of vaccine services. Resolving early implementation problems (51) is a priority for the groups of children that are the focus of this paper.

Despite high rates of completed vaccinations among children receiving care or support, timeliness remains suboptimal. Any deviations from immunisation schedules may compromise both individual’s protection and population (herd) immunity. Vaccinations administered before the recommended intervals may induce a reduced immune response, whereas longer intervals may leave children under protected (2). Therefore, interventions to improve the timeliness of vaccinations such as educational, clinical and policy interventions should be put in place. Overall, it is very encouraging to see high rates of immunisation coverage throughout Wales, although healthcare policies should be updated to promote more timely vaccination to ensure optimal protection for these vulnerable children.

#### Data availability statement

Whilst we were able to utilise the NCCHD which covers the whole population of Wales, there is currently no unified system in place which would allow an equivalent analysis in England or Scotland. Although Public Health Scotland publish annual uptake rates by age and deprivation (52) and previous work in England has been limited to primary health care records from 100 general practice [Oxford-Royal College of General Practitioners (RCGP) Research and Surveillance Centre (RSC)], (2) this is not to the same extent as the work conducted here.

## Conclusion

Our study presents the encouraging findings that looked after children in Wales are more likely to be up-to-date with immunisations than the general child population. This finding runs counter to prior studies and may suggest some success in policies designed to support looked after children’s health needs. Our study also presents findings on a less understood group: children receiving services at home under a care and support plan, including those on the Child Protection Register. Perhaps surprisingly, this group, too, are more likely to be up-to-date with immunisations. This may suggest a “protective” factor related to the higher level of state intervention, including having an allocated social worker. Nonetheless, all groups of children receiving support under a care and support plan were more likely to have experienced early or late vaccinations, demonstrating that more inter-disciplinary co-ordination and planning is still needed to improve these outcomes.

## Data availability statement

The data analysed in this study is subject to the following licenses/restrictions: data may be obtained from a third party and are not publicly available. The data used in this study is available from the Secure Anonymised Information Linkage (SAIL) Databank at Swansea University, Swansea, UK, which is part of the national e-health records research infrastructure for Wales. All proposals to use SAIL datasets must comply with HIRU’s information governance policy and are subject to review by an independent Information Governance Review Panel (IGRP). Before data can be accessed, approval must be given by the IGRP. Requests to access these datasets should be directed to www.saildatabank.com/application-process.

## Ethics statement

Ethical review and approval was not required for the study on human participants in accordance with the local legislation and institutional requirements. Written informed consent from the participants’ legal guardian/next of kin was not required to participate in this study in accordance with the national legislation and the institutional requirements.

## Author contributions

AL performed the analysis, with LG, MP, and HB interpreting the results. GB, LG, and HB drafted the first iteration of the manuscript. All authors contributed to the conception and design of this work, critically reviewed the manuscript, provided important intellectual input, and approved the final version and agreed to be accountable for their contributions.

## Funding

The CASCADE partnership receives infrastructure funding from Health and Care Research Wales (517199). This study uses anonymised data held in the Secure Anonymised Information Linkage (SAIL) Databank, which is part of the national population data research infrastructure for Wales.

## Conflict of interest

The authors declare that the research was conducted in the absence of any commercial or financial relationships that could be construed as a potential conflict of interest.

## Publisher’s note

All claims expressed in this article are solely those of the authors and do not necessarily represent those of their affiliated organizations, or those of the publisher, the editors and the reviewers. Any product that may be evaluated in this article, or claim that may be made by its manufacturer, is not guaranteed or endorsed by the publisher.
