# Supplementary figures and images for: Immunisation status of children receiving care and support in Wales: a national data linkage study
Source: Front Public Health. 2023 Jul 31;11:1231264. doi: 10.3389/fpubh.2023.1231264 (PMC10423803; doi:10.3389/fpubh.2023.1231264)

**APPENDIX 1: Population Cohort comparison group**


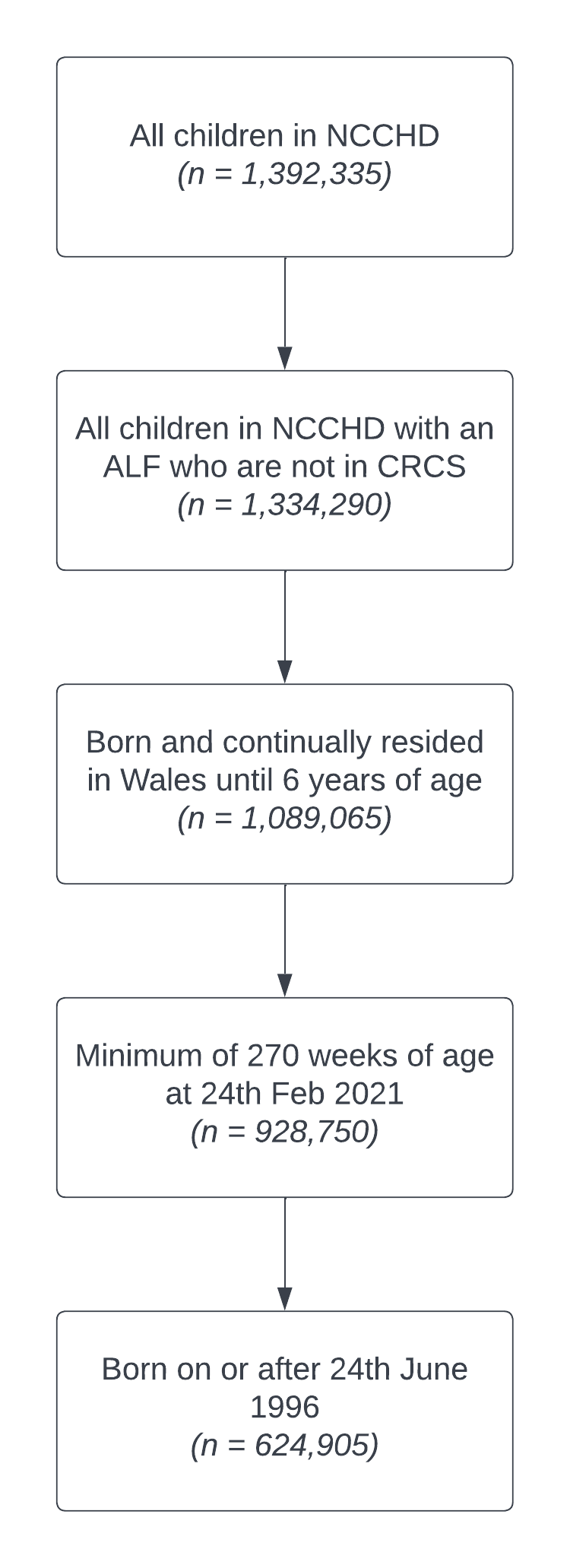

Supplement: Supplementary file 1 [file Table_1.DOCX]
