# Supplementary material for: Immunisation status of children receiving care and support in Wales: a national data linkage study
Source: Front Public Health. 2023 Jul 31;11:1231264. doi: 10.3389/fpubh.2023.1231264 (PMC10423803; doi:10.3389/fpubh.2023.1231264)
Supplement: Supplementary file 2 [file Table_2.DOCX]

**APPENDIX 2: Timely, early, delayed or no-immunisation as recorded in CRCS records.**

|  | **Population** | **CRCS cohort** | **Never CPR not CLA** | **Never CPR not CLA** | **Ever CLA** |
| --- | --- | --- | --- | --- | --- |
| Total | 624905 | 24540 | 12480 | 6225 | 5840 |
| **On time** | | | | |  |
| At least one vaccine on time | 603345  (96.5%) | 21225  (86.5%) | 10785  (86.4%) | 5375  (86.3%) | 5060  (86.6%) |
| All vaccines on time | 183425  (29.4%) | 4130  (16.8%) | 2165  (17.3%) | 1210  (19.4%) | 755  (12.9%) |
| **Early** | | | | |  |
| At least one vaccine early | 32365  (5.2%) | 5455  (22.2%) | 2805  (22.5%) | 1350  (21.7%) | 1300  (22.3%) |
| All vaccines early | 0  (0%) | 1345  (5.5%) | 680  (5.4%) | 425  (6.8%) | 240  (4.1%) |
| **Delayed** | | | | |  |
| At least one vaccine delayed | 391835  (62.7%) | 17500  (71.3%) | 8745  (70.1%) | 4190  (67.3%) | 4565  (78.2%) |
| All vaccines delayed | 2930  (0.5%) | 530  (2.2%) | 230  (1.8%) | 115  (1.8%) | 185  (3.2%) |
| **Missing** | | | | |  |
| At least one vaccine missing | 81340  (13.0%) | 2055  (8.4%) | 1255  (10.1%) | 450  (7.2%) | 350  (6.0%) |
| All vaccines missing | 10510  (1.7%) | 65  (0.3%) | 40  (0.3%) | 15  (0.2%) | 10  (0.2%) |
|  |  |  |  |  |  |
| **Descriptives** |  |  |  |  |  |
| **On time** |  |  |  |  |  |
| Mean number of vaccines on time | 4.4 | 3.4 | 3.4 | 3.4 | 3.3 |
| Median number of vaccines on time | 5 | 3 | 3 | 3 | 3 |
| Standard deviation of vaccines on time | 1.6 | 2.1 | 2.1 | 2.1 | 2.0 |
| 1st quartile of vaccines on time | 4 | 2 | 2 | 2 | 2 |
| 2nd quartile of vaccines on time | 5 | 3 | 3 | 3 | 3 |
| 3rd quartile of vaccines on time | 6 | 5 | 5 | 5 | 5 |
| **Early** | | | | | |
| Mean number of vaccines early | 0.1 | 0.9 | 0.9 | 0.9 | 0.8 |
| Median number of vaccines early | 0 | 0 | 0 | 0 | 0 |
| Standard deviation of vaccines early | 0.3 | 1.8 | 1.8 | 1.9 | 1.8 |
| 1st quartile of vaccines early | 0 | 0 | 0 | 0 | 0 |
| 2nd quartile of vaccines early | 0 | 0 | 0 | 0 | 0 |
| 3rd quartile of vaccines early | 0 | 0 | 0 | 0 | 0 |
| **Delayed** | | | | | |
| Mean number of vaccines delayed | 1.2 | 2.0 | 1.9 | 1.8 | 2.3 |
| Median number of vaccines delayed | 1 | 1 | 1 | 1 | 2 |
| Standard deviation of vaccines delayed | 1.3 | 1.8 | 1.8 | 1.8 | 1.9 |
| 1st quartile of vaccines delayed | 0 | 0 | 0 | 0 | 1 |
| 2nd quartile of vaccines delayed | 1 | 1 | 1 | 1 | 2 |
| 3rd quartile of vaccines delayed | 2 | 3 | 3 | 3 | 4 |
| **Missing** | | | | | |
| Mean number of vaccines missing | 0.4 | 0.2 | 0.2 | 0.1 | 0.1 |
| Median number of vaccines missing | 0 | 0 | 0 | 0 | 0 |
| Standard deviation of vaccines missing | 1.1 | 0.6 | 0.7 | 0.6 | 0.5 |
| 1st quartile of vaccines missing | 0 | 0 | 0 | 0 | 0 |
| 2nd quartile of vaccines missing | 0 | 0 | 0 | 0 | 0 |
| 3rd quartile of vaccines missing | 0 | 0 | 0 | 0 | 0 |
